# Gene expression of muscarinic, tachykinin, and purinergic receptors in porcine bladder: comparison with cultured cells

**DOI:** 10.3389/fphar.2013.00148

**Published:** 2013-11-28

**Authors:** Forough Bahadory, Kate H. Moore, Lu Liu, Elizabeth Burcher

**Affiliations:** ^1^Department of Pharmacology, School of Medical Sciences, University of New South WalesSydney, NSW, Australia; ^2^Detrusor Muscle Laboratory, St. George Hospital, University of New South WalesKogarah, NSW, Australia

**Keywords:** smooth muscle, urothelium, suburothelium, myofibroblasts, muscarinic receptors, tachykinin receptors, purinergic receptors, cell culture

## Abstract

Urothelial cells, myofibroblasts, and smooth muscle cells are important cell types contributing to bladder function. Multiple receptors including muscarinic (M_3_/M_5_), tachykinin (NK_1_/NK_2_), and purinergic (P2X_1_/P2Y_6_) receptors are involved in bladder motor and sensory actions. Using female pig bladder, our aim was to differentiate between various cell types in bladder by genetic markers. We compared the molecular expression pattern between the fresh tissue layers and their cultured cell counterparts. We also examined responses to agonists for these receptors in cultured cells. Urothelial, suburothelial (myofibroblasts), and smooth muscle cells isolated from pig bladder were cultured (10–14 days) and identified by marker antibodies. Gene (mRNA) expression level was demonstrated by real-time PCR. The receptor expression pattern was very similar between suburothelium and detrusor, and higher than urothelium. The gene expression of all receptors decreased in culture compared with the fresh tissue, although the reduction in cultured urothelial cells appeared less significant compared to suburothelial and detrusor cells. Cultured myofibroblasts and detrusor cells did not contract in response to the agonists acetylcholine, neurokinin A, and β,γ-MeATP, up to concentrations of 0.1 and 1 mM. The significant reduction of M_3_, NK_2_, and P2X_1_ receptors under culture conditions may be associated with the unresponsiveness of cultured suburothelial and detrusor cells to their respective agonists. These results suggest that under culture conditions, bladder cells lose the receptors that are involved in contraction, as this function is no longer required. The study provides further evidence that cultured cells do not necessarily mimic the actions exerted by intact tissues.

## Introduction

Interactions between various neurochemicals and receptors are essential for normal sensory and motor function in the urinary bladder (Birder, 2013; Burnstock, 2013). The main neurotransmitters involved in detrusor muscle contraction and relaxation are acetylcholine (ACh) and noradrenaline (NA), respectively, (Ochodnicky et al., 2013). Other mediators such as adenosine triphosphate (ATP), nitric oxide (NO), and tachykinins are also involved in bladder function, and are of importance in stimulating afferent signaling pathways (Ishizuka et al., 1995; Otomo et al., 1999; Whitbeck et al., 2007).

In recent years it has become apparent that the bladder lining (urothelium and lamina propria, collectively known as the mucosa) may play a significant role in the initiation and propagation of afferent signals during bladder filling (Fry et al., 2007). The mucosa contains various cell types, of major interest being spindle shaped cells known as myofibroblasts, mainly located in the suburothelium (Cheng et al., 2011). These cells are connected by gap junctions (connexin 43), and localized in a close proximity to C fiber nerves (Wiseman et al., 2003) and other cell types in the bladder wall (McCloskey, 2010). The function of myofibroblasts is not fully understood. However, studies have shown that these cells can elicit spontaneous electrical currents, and respond to exogenous agents such as ATP via P2Y_6_ receptors (Wu et al., 2004). Furthermore, our group has shown the potent contractility of bladder suburothelium in response to the tachykinin and muscarinic receptor agonists; it was suggested that myofibroblasts might be the cell type responsible for suburothelial contraction (Sadananda et al., 2008, 2012).

Porcine bladder is a well recognized model for the human bladder (Templeman et al., 2003; Kumar et al., 2004; Sadananda et al., 2008). Previously, our group illustrated that three distinct cell types: urothelial, myofibroblast, and detrusor muscle, can be cultured from porcine bladder (Cheng et al., 2011). The cells were distinguished on the basis of morphological, immunological, and pharmacological characterization and were able to release ATP in response to stretch (Cheng et al., 2011), but the differences in gene expression between various cell types in bladder have not been studied.

Our primary aim was to examine the gene expression and distribution pattern of the principal receptors mediating contraction of the urinary bladder: muscarinic M_3_, tachykinin NK_2_, and purinergic P2X_1_ receptors. We also studied expression of M_5_ and P2Y_6_ receptors. We compared the expression in tissue from three bladder regions: urothelium, suburothelium (the dissected mucosa minus urothelium), and detrusor, with their cultured cell counterparts. We hypothesized that the cultured cells would show similarities to the fresh tissue with respect to gene expression, and that each cultured cell type should be characterized by a different set of genetic markers. This study also examined any phenotypic alteration of the cells in culture, as reported for smooth muscle cells (Nair et al., 2011; Huber and Badylak, 2012), and determined the reliability of primary cultured cells in investigating the function of isolated bladder cell types. We also examined the functional properties of the cultured cells, using agonists for muscarinic M_3_, tachykinin NK_2_, and purinergic P2X_1_ receptors, in suburothelium and detrusor. To our knowledge this is the first report of gene expression for those receptors in the porcine bladder.

## Methods and materials

### Tissue preparation and cell culture

Female pigs (6–9 months, ~55 kg), were sacrificed at a local abattoir. Bladders were collected and transported on ice to the laboratory. Any damaged or inflamed bladders were discarded. Fat tissue was removed and bladders were rinsed with Krebs-Henseleit solution.

For cell culture and molecular studies, the urothelial tissue (UT) was scraped off using a scalpel blade. To remove any residual urothelium, trypsin (0.25% with EDTA) was then applied to the luminal surface for 5 min at 37°C, neutralized with RPMI culture medium and rinsed with Krebs-Henseleit solution. The bladder (minus urothelium) was then dissected into two layers, the mucosa and the detrusor (DT). The mucosa was further dissected under a microscope to remove the residual muscle bundles and blood vessels, as described (Sadananda et al., 2008), leaving a thin layer of suburothelium (ST) used in this study. We did not attempt to separate myofibroblasts from fibroblasts.

One half of these fresh dissected tissues was snap frozen and stored at −80°C overnight for molecular studies. The remainder was minced, and treated with 0.25% trypsin-EDTA with 0.15% collagenase type II for 30 min at 37°C, followed by neutralization with RPMI culture medium. The cells cultured into three populations: urothelial cells (UC), suburothelial cells (SC) which included myofibroblasts, and detrusor muscle cells (DC), as described (Cheng et al., 2011). Cells were harvested after 10–14 days in primary culture.

### Fluorescent immunohistochemistry and immunocytochemistry

Additional segments of porcine bladder dome were fixed in Zamboni's solution overnight at 4°C. The segments were washed in dimethyl sulfoxide (DMSO, 3 × 10 min), rinsed in phosphate buffered saline (PBS, 0.1M, pH = 7.4), and then immersed in 30% sucrose in PBS overnight at 4°C. Paraffin sections were processed (dewaxation and rehydration), and preincubated with hydrogen peroxide (3%) for 5–8 min, followed by incubation overnight at room temperature with primary marker antibodies: AE1/AE3 (M3515), vimentin (M0725), or α-smooth muscle actin (M0851) (α-SMA, 1:500 dilution with 2% goat serum). Immunostaining with c-kit (SC 13508) was also attempted. Thereafter, slides were rinsed (3 × 5 min) with PBS and incubated with the secondary antibody (FITC-conjugated anti-mouse IgG antibody, 1:100 in 2% goat serum) at room temperature for 1 h. The sections were washed with PBS and coverslipped with mounting medium (ProLong^®^ Gold Antifade Reagent with DAPI, Life technologies). In each run, negative controls (no primary antibody), and positive controls (human colon) were also included. The fields were observed using a fluorescence microscope (EVOS fl: filter GFP, 40× magnification).

Cultured cells were also immunostained to confirm their identity. Ethanol (100%) was added to culture wells for 15 min at room temperature to fix the cells, which were then permeabilized with 0.1% Triton X-100 in PBS for 10 min. The primary and secondary antibodies were applied as above.

### Masson's trichrome blue stain

Sections adjacent to those used fluorescent immunohistochemistry were stained with Masson's trichrome to delineate connective tissue from smooth muscle and from urothelial cells. In brief, sections were re-hydrated with ethanol and then distilled water, stained in hematoxylin for 10 min, and rinsed in warm tap water for 10 min. They were covered by Biebrich scarlet-acid fuchsin solution for 15 min followed by washing in distilled water. The sections were incubated in phosphomolybdic-phosphotungstic acid solution for 15 min or until the collagen was not red, then covered by aniline blue solution for 5–10 min. They were washed in distilled water briefly, and were differentiated using 1% acetic acid solution for 2–5 min. After rinsing in water, the sections were dehydrated, cleared in xylene and mounted.

### RNA extraction and RT-PCR

RNA was extracted from thawed bladder tissues (*n* = 10–14) and 80% confluent cultured cells by the TRIzol method (Invitrogen). Total RNA (2 μg) was subjected to single strand cDNA synthesis using a SuperScript™ III First-Strand Synthesis System. The genes under investigation encoded for the muscarinic M_3_ and M_5_ receptors, the tachykinin receptors (NK_1_ and NK_2_), and the purinergic P2X_1_ and P2Y_6_ receptors. The gene sequences of interest were derived from the pig genome (National Centre for Biotechnology Information, NCBI), and primers were designed using Primer 3.0 software (Table 1). The gene sequence for NK_1_ receptor in porcine genome is not published in NCBI, therefore, the primers for the NK_1_ receptor were derived from sequences conserved across a broad range of mammalian species (human, cow, dog, and mouse). Gene expression was determined by quantitative real-time polymerase chain reaction (qRT-PCR) using the KAPA™ SYBR^®^ FAST kit as described (Liu et al., 2011; Dai et al., 2012). GAPDH was used as the housekeeping gene (HKG), and mRNA from an intact pig bladder tissue was used as the internal calibrator.

**Table 1:** **Oligonucleotide primers used in the real-time PCR studies**.

**Gene**	**Protein**	**Primer sequence (5′–3′), forward/reverse**	**Product size (bp)**
*pChrm*_*3*_	M_3_	GCCATCTACTCCATCGTGCT/ CTCTTCTGGGCTTGCAGTTT	152
*pChrm*_*5*_	M_5_	GTCTGAGCCCACCATCACTT	248
		AAGAGGCTTGGTTCCTTTCC	
*pTACR*_1_^*^	NK_1_	TACTCCATGACGGCTGTGG/ ATCTTGTTGGGATGCTCTGG	212
*pTACR*_*2*_	NK_2_	CCTGTGATGTGGTGACTGATG/ GCCAGGTTGACGATGAAGTAG	204
*pP2RX_1_*	P2X_1_	TCATCAAGAACAGCATCAGCTT/ CAGTCCAGGTCACAGTTCCA	232
*pP2RY_6_*	P2Y_6_	CCACCCACTACATGCCCTAT	217
		GTGATGTGGAAAGGCAGGAA	
*pGapdh*	GAPDH	ACCCAGAAGACTGTGGATGG/ CCCCAGCATCAAAGGTAGAA	346

Primer sequences are based on the pig genome except for pTACR_1_

(*) which is based on the sequences in other mammalian species.

Data were expressed as mean ± s.e.m. The mRNA level for each gene was expressed as fold change relative to GAPDH and the calibrator using the formula:

Fold change = 2^−ΔΔCt^,

where Δ Δ C_*t*_ = [C_*t*_(target) − C_*t*_(HKG)]sample − [C_*t*_(target) − C_*t*_(HKG)]calibrator (Pfaffl, 2001). For each gene, One-Way ANOVA followed by Bonferroni's multiple comparison tests was used to compare the mean values between tissues and cells, as well as between different tissues. For all studies, the *n*-value was taken as the number of pigs.

### Functional studies with cultured cells

Cultured suburothelial and detrusor muscle cells (10–14 days) were exposed to PBS (control), ACh (10 μM–1 mM), carbachol (1 μM–10 mM), NKA (1–100 μM), β,γ-MeATP (1 μM–1 mM), as well as KCl (80 and 160 mM), and photographed every 5 s up to 5 min. The changes in the length and width of the cells were measured using ImageJ (National Institutes of Health, USA).

## Materials

Cell culture products, TRIzol RNA purification reagent and SuperScript™ III First-Strand cDNA Synthesis System were obtained from Invitrogen (Mt Waverley, Australia). DNase enzymes were from Promega (Madison, USA). Primers, RNALater and the KAPA™ SYBR^®^ FAST qPCR reagents were from GeneWorks (Adelaide, Australia). Primary antibodies were from DakoCytomation (Campbellfield, Australia). The c-kit antibody was obtained from Santa Cruz Biotechnology, Dallas, Texas, USA. FITC-conjugated anti-mouse IgG was from Abcam (Waterloo, Australia). ACh, carbachol, NKA, and β,γ-MeATP were purchased from Sigma-Aldrich (Sydney, Australia). NKA was initially dissolved in 0.01M acetic acid with 1% β-mercaptoethanol, and other agonists were dissolved in Krebs-Henseleit solution.

## Results

### Fluorescent immunohistochemistry and immunocytochemistry

**Figure 1:**
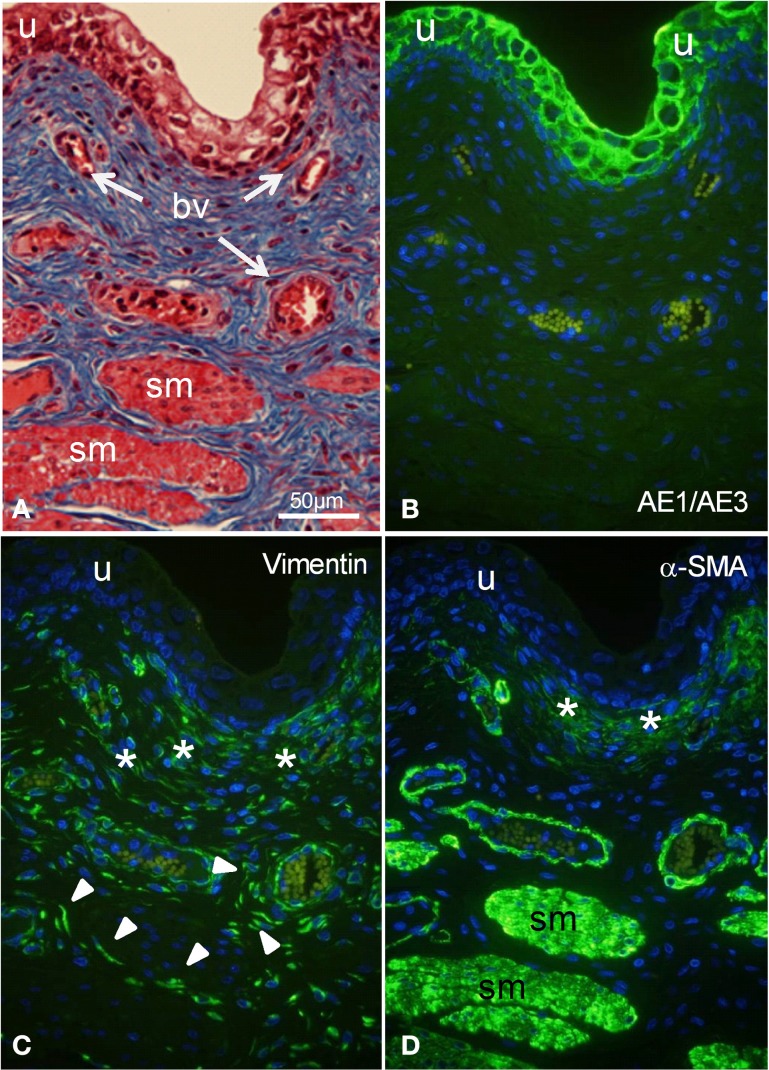
**(A)** Histological staining **(A)** and fluorescent immunostaining **(B–D)** of intact segments of porcine bladder. **(A)** Masson's staining showing urothelium (u), smooth muscle bundles (sm), and blood vessels (bv). **(B)** Immunocytochemical characterization with AE1/AE3 (cytokeratin marker) shows staining of urothelium only. **(C)** Immunostaining with vimentin shows labeling of large spindle-shaped cells (arrowheads) and a cell layer (^*^) under the urothelium. **(D)** Immunostaining with α-SMA (smooth muscle marker) shows labeling of smooth muscle bundles, the outer perimeter of blood vessels as well as part of a cell layer (^*^, suburothelium) under the urothelium. All cells were double stained with DAPI (cell nuclei, blue). All panels are shown at the same magnification. Bar = 50 μm.

Adjacent sections of whole porcine bladder were stained histologically with Masson's (Figure 1A) to reveal histological features, and immunostained with marker antibodies (Figures 1B–D). The urothelium was stained by the cytokeratin marker AE1/AE3 (Figure 1B) but not by vimentin (Figure 1C) or by α-SMA (Figure 1D). A cell layer 40–50 μm thick under the urothelium was stained moderately by both vimentin and α-SMA. Blood vessels in the lamina propria were stained strongly by α-SMA and weakly by vimentin. Detrusor muscle bundles were stained strongly by α-SMA and not at all by vimentin. However, large spindle shaped cells throughout the lamina propria were stained only by vimentin.

**Figure 2:**
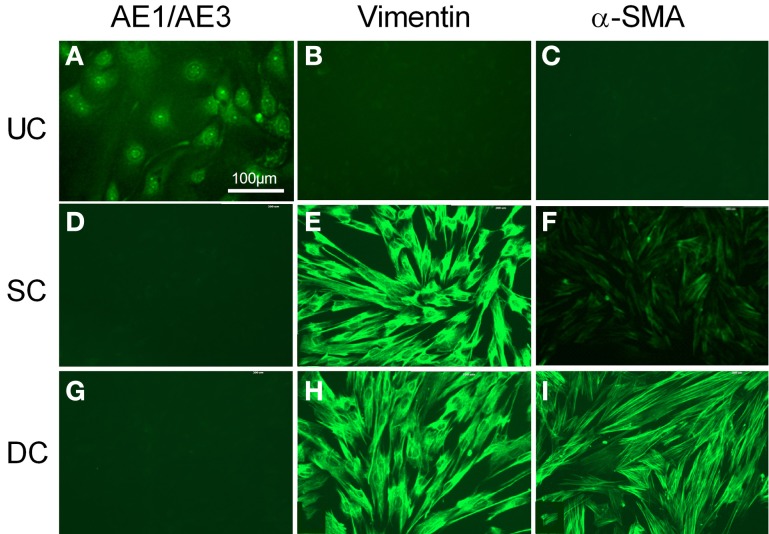
**Fluorescent immunocytochemical characterization of cultured urothelial cells (A–C), suburothelial cells (D–F), and detrusor smooth muscle cells (G–I)**. Cells **(A,D,G)** were immunostained by AE1/AE3 (cytokeratin marker), **(B,E,H)** by vimentin, and **(C,F,I)** by α-SMA (smooth muscle marker). Cells were double stained with DAPI (cell nuclei, blue). All panels are shown at the same magnification. Bar = 100 μm.

Cultured urothelial cells were stained by AE1/AE3 (Figure 2A), but showed no immunoreactivity for vimentin or α-SMA (Figures 2B,C). The morphology of cultured suburothelial cells (myofibroblasts) and detrusor muscle cells was very similar, and neither showed any immunoreactivity to AE1/AE3 (Figures 2D,G). Suburothelial cells displayed strong immunoreactivity to vimentin (Figure 2E), but weak immunoreactivity to α-SMA (Figure 2F). Cultured detrusor muscle cells, on the other hand, showed strong staining for both vimentin and α-SMA (Figures 2H,I). Sections in which the primary antibody was omitted (negative controls) showed no autofluorescence except for erythrocytes (not shown). Staining with the c-kit antibody was unsuccessful in the pig bladder.

### Gene expression

**Figure 3:**
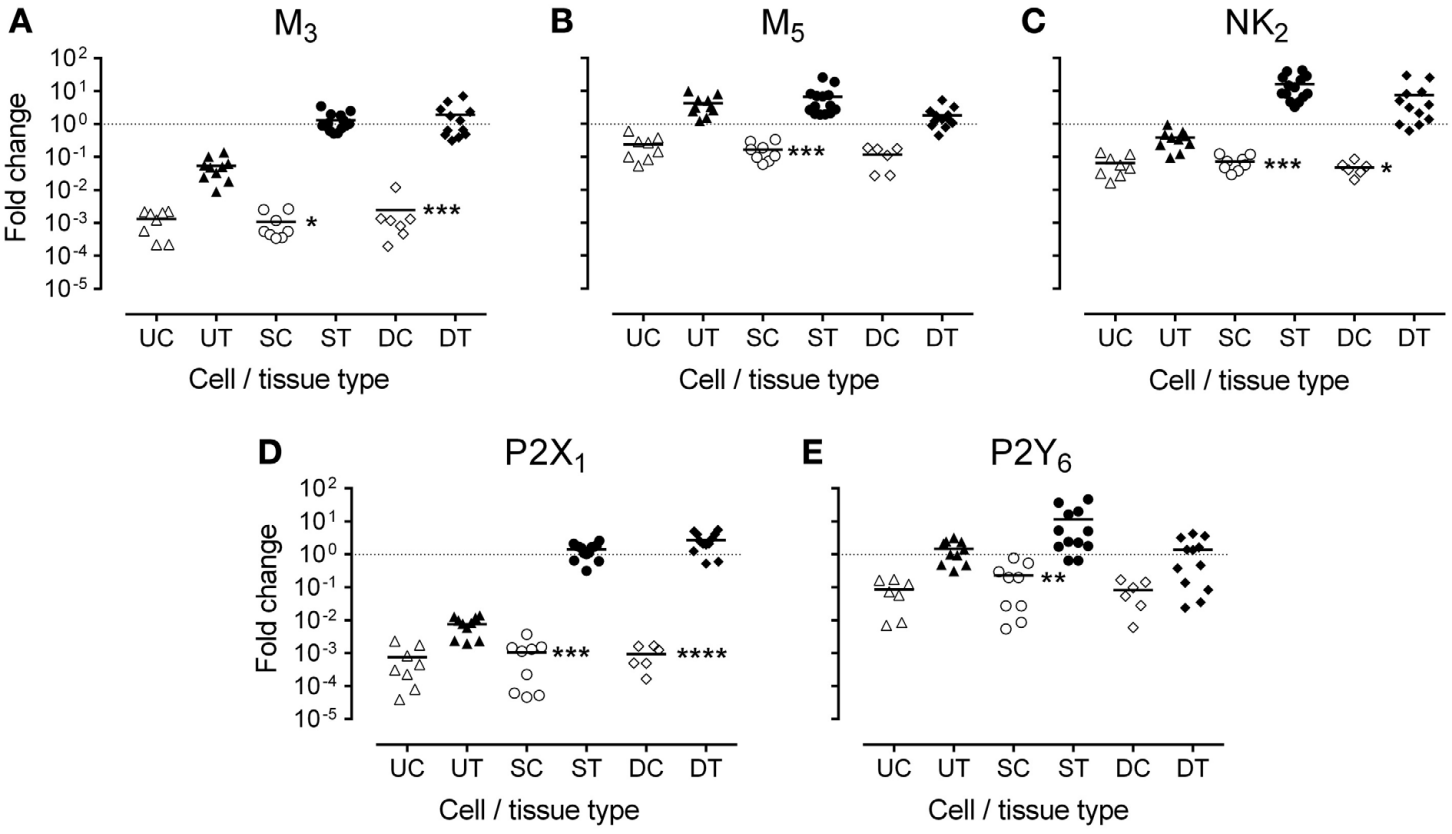
**Expression of mRNA for (A) muscarinic (M_3_), (B) muscarinic (M_5_), (C) tachykinin (NK_2_), (D) purinergic (P2X_1_), and (E) purinergic (P2Y_6_) receptors, in urothelium, suburothelium, and detrusor tissue (UT, ST, DT) and cultured cells (UC, SC, DC) in porcine bladder (*n* = 6–14)**. Data are expressed as fold change relative to GAPDH and the calibrator. The bar represents the mean, and the dotted line is drawn at 1. Significant reductions in gene expression in cell culture compared with corresponding native tissue were observed. ^*^P < 0.05; ^**^P < 0.01; ^***^P < 0.001; ^****^P < 0.0001 (One-Way ANOVA followed by Bonferroni's multiple comparisons test).

In fresh frozen tissues, all transcripts, except M_5_, were highly expressed in detrusor and suburothelium, but often poorly expressed in the urothelium (Table 2 and Figure 3). Expression of the muscarinic M_3_ receptor was similarly high in both suburothelium and detrusor, and extremely low in urothelium. Muscarinic M_5_ expression was fairly similar in suburothelium and urothelium, but lower in detrusor. The expression order for the NK_2_ receptor was suburothelium > detrusor > urothelium, whereas the purinergic P2X_1_ receptor was expressed in detrusor > suburothelium, but not in urothelium. P2Y_6_ was highly expressed in suburothelial tissues > urothelium = detrusor.

**Table 2:** **Gene expression level for receptor transcripts in urothelium, suburothelial, and detrusor tissues**.

**Tissue type**	**Receptors**				
	**M**_3_****	**M**_5_****	**NK**_2_****	**P2X**_1_****	**P2Y**_6_****
Urothelium	0.05	4.22	0.38	~0.00	1.46
Suburothelium	1.29^*^	6.69	16.1^***^	1.45^***^	11.5^**^
Detrusor	1.89^***^	1.79^††^	7.46^*^^†^	2.69^****^,^†††^	1.36^††^

Data expressed as fold change compared to the calibrator and GAPDH.

* P < 0.05

** P < 0.01

*** P < 0.001

**** P < 0.0001, compared with urothelium.

† P < 0.05

†† P < 0.01

††† P < 0.001, compared with suburothelium (One-Way ANOVA, followed by Bonferroni's multiple comparisons test).

In general, expression of the receptor transcripts decreased in cell culture compared with that in the corresponding fresh tissues (Figure 3). For M_3_, NK_2_, and P2X_1_, in particular, this reduction was highly significant with respect to cultured suburothelium and detrusor. In contrast, the reduction in cultured urothelial cells was not significant for any transcript. Notably, no expression of the tachykinin NK_1_ receptor was observed in any tissues or cultured cells of the pig bladder.

### Functional studies

Cultured suburothelial and detrusor cells did not respond to ACh and the cholinergic agonist carbachol, nor to NKA, and the P2X_1_ selective agonist β, γ-MeATP, at any concentration (up to 0.1 mM for NKA, 1 mM for ACh, and β, γ-MeATP, or 10 mM for carbachol) or time point up to 5 min (data not shown). In contrast, when KCl (80 mM) was applied to cultured smooth muscle cell, 60–70% cells showed visible responses to KCl. The detachment of a few cells from the well surface at this concentration was also observed (data not shown). The higher concentrations of KCl (160 mM) resulted in detachments of more cells. These results suggested that cultured cells can contract in response to KCl. Moreover, suburothelial and detrusor strips contracted in a concentration-dependent manner to ACh, NKA, and β, γ-MeATP, and KCl (data not shown), verifying the efficacy of the drug solutions.

## Discussion

Here, we have successfully cultured three different cell types from porcine bladders [as described in Cheng et al. (2011)] with an aim to study gene expression of some key receptors in bladder contraction and afferent signaling. We hypothesized that each cell type may express different genes according to their function in the bladder. This study showed that the expression of muscarinic M_3_, tachykinin NK_2_, and purinergic P2X_1_ receptors was high in fresh detrusor and suburothelium, and lower in fresh urothelial cells. Their corresponding receptor proteins have been demonstrated to have contractile functions in the urinary bladder (Mussap et al., 1996; Vial and Evans, 2000; Fetscher et al., 2002; Yoshimura et al., 2008). However, our major finding was that expression of these three genes was significantly decreased in primary culture. We were not able to detect the NK_1_ transcript in the porcine bladder, despite using primers derived from sequences conserved across a broad range of mammalian species, which showed a positive NK_1_ product in human colon. This is unexpected given the prominent role of the NK_1_ receptor in micturition in other mammalian bladders (Yoshimura et al., 2008). Overall, our original hypothesis, that urothelial cells, smooth muscle cells and myofibroblasts (suburothelial cells) should be characterized by a different set of genetic markers, was not substantiated. It is worth noting that the detrusor and suburothelium would contain various cell types (e.g., nerves, blood vessels, etc) in addition to smooth muscle and myofibroblasts, respectively. Thus, the transcripts might have been originally associated with a variety of cell types that did not grow in culture. Furthermore, the expression level of a transcript may not be related to the expression level of its associated protein.

The function of muscarinic M_5_ receptors in bladder is unclear due to the continuing lack of a selective receptor antagonist, but they are known to be associated with the urothelium (Mansfield et al., 2005). Here, we found that these receptors were associated with all three cell populations, which was consistent with our previous finding in the human bladder, where M_5_ receptor transcript was expressed in both detrusor and mucosa (Mansfield et al., 2005). Unlike M_3_ receptor, the loss M_5_ expression in cell culture was rather moderate.

Muscarinic receptors respond to the parasympathetic neurotransmitter acetylcholine, to contract the detrusor, resulting in bladder emptying. The main receptor involved in the bladder detrusor muscle contraction is the M_3_ receptor (Choppin et al., 1998; Sellers et al., 2000; Chess-Williams et al., 2001), the major target for the treatment of lower urinary tract symptoms such as urinary frequency and urgency. Likewise, tachykinin peptides including NKA are able to contract the detrusor predominately via NK_2_ receptors (Bushfield et al., 1995; Sadananda et al., 2008), and also have sensory functions in bladder (Maggi et al., 1988, 1991). ACh and NKA can also contract the suburothelium (Sadananda et al., 2008). It is assumed that these agonists cause detrusor contraction directly via smooth muscle cells, but the mechanisms underlying suburothelial contraction are less clear. It has previously been hypothesized that suburothelial myofibroblasts may be the cell types causing contraction of the porcine bladder mucosa (Sadananda et al., 2008, 2009), but histological staining of mucosal and suburothelial strips reveals variable amounts of thin smooth muscle bands, of different appearance to detrusor muscle bundles. As expected, both suburothelial and detrusor tissue strips contracted to the muscarinic and tachykinin agonists, suggesting the presence of M_3_ (Sellers et al., 2000) and NK_2_ (Sadananda et al., 2008) receptors in the native tissue. However, the cultured cells of both suburothelium and smooth muscle were completely unable to contract to either ACh or NKA, correlating with the loss of receptor expression in cell culture. However, it should be noted that downregulated expression of receptor transcripts in cultured conditions is not necessarily reflected by the lack of contractile response of the cells to the receptor agonists. Other mechanisms may be involved. For instance, cell responses in the culture may be affected by the way of cell attachment to the plastic well surface. Individual cells in the culture lack of synergistic activities as a consequence of the loss of cell-to-cell communication. Furthermore, the expression contractile elements may also be downregulated, or contractile protein phosphorylation is reduced during culture. Further studies to elucidate the mechanisms should be of interest; however, they are beyond the scope of this report. It should be noted that muscarinic receptors can mediate smooth muscle proliferation in cell culture and may contribute to bladder remodeling (Arrighi et al., 2013).

As seen for ACh and NKA, the cultured suburothelial and smooth muscle cells failed to contract to the selective P2X_1_ agonist β, γ-MeATP, in accordance with the loss of P2X_1_ receptors in culture. The endogenous purinergic agonist ATP exerts a broad range of functions in bladder by binding to P2X and P2Y receptors (Burnstock, 2007, 2013). ATP has neural functions and is a parasympathetic co-transmitter contributing to bladder emptying via P2X_1_ mediated detrusor contraction (Chancellor et al., 1992; Akino et al., 2008). The most abundant P2X receptor in the mammalian bladder, P2X_1_ (Longhurst et al., 1996), is widely accepted to have excitatory effects on detrusor smooth muscle. Non-neuronal ATP can be released from urothelial and other cell types, and we have previously demonstrated that ATP release is significantly higher in urothelial and myofibroblast (suburothelial) cultures than in detrusor culture (Cheng et al., 2011). Similarly, more ATP is released from suburothelial than detrusor strips (Sadananda et al., 2009; Yoshida et al., 2009). ATP released from bladder lining can stimulate P2X_3_ receptors on the nerves (Burnstock, 2007), which activates afferent signaling and thus, elicit the sensation of bladder fullness to the brain. Therefore, the role of ATP in bladder mucosa is more associated with the sensation of bladder filling rather than emptying.

Our studies showed that P2Y_6_ was expressed in all bladder layers, particularly in suburothelial tissue, supporting the previous observation that P2Y_6_ is a predominant purinoceptor in bladder suburothelium, particularly on myofibroblasts (Sui et al., 2006). The role of P2Y_6_ in bladder is not clear, but may be involved in bladder relaxation (Tong et al., 1997). Note that activation of P2Y_6_ receptors by UDP enhanced ATP-mediated contractile force in mouse whole bladder, suggesting synergism between P2X_1_ and P2Y_6_ receptors (Yu et al., 2013). Further studies to uncover the underlying purinergic mechanisms are warranted.

The unresponsiveness of cultured suburothelial and detrusor muscle cells to the muscarinic, tachykinin, and purinergic agonists demonstrated altered functionality in cell culture. The phenotypic alteration of smooth muscle cells in culture has previously been reported (Nair et al., 2011; Huber and Badylak, 2012). The former group suggested that proliferation reduces the expression of specific genes and proteins involved in contraction of smooth muscle cells, an effect observed by day 4 in cell culture (Nair et al., 2011). Thus, even in primary culture, cells do not appear to express those receptor proteins which are not required for their function in the culture. Additionally our data showed that the gene expression in all cell types reached the same pattern in the culture. This may indicate that different cell types, which were differentiated in the native tissue, may undergo myogenic conversion after 2 weeks in the culture. Several studies have reported the conversion of fibroblasts, myofibroblasts and muscle cells depending on the experimental conditions (Darby et al., 1990; Salvatori et al., 1995; Chiavegato et al., 1999; Darby and Hewitson, 2007).

In conclusion, this study showed that the expression of the receptor transcripts involved in muscle contraction (M_3_, NK_2,_ and P2X_1_) decreased in the primary cultured cells (SC and DC). The unresponsiveness of cultured cells to muscarinic, tachykinin, and purinergic receptor agonists correlates with the low expression of receptor transcripts. This suggests the reduction in expression of not only the transcripts, but also of their related proteins. These results provide evidence that cultured cells do not necessarily mimic the actions exerted by intact tissues.

## Author contributions

Forough Bahadory conducted the experiments, analyzed the data, and wrote the manuscript. Kate H. Moore provided intellectual input. Lu Liu provided intellectual input, technical and analytical support and contributed to the manuscript. Elizabeth Burcher provided intellectual input and wrote the manuscript.

### Conflict of interest statement

The authors declare that the research was conducted in the absence of any commercial or financial relationships that could be construed as a potential conflict of interest.
