# Supplementary material for: Gene expression of muscarinic, tachykinin, and purinergic receptors in porcine bladder: comparison with cultured cells
Source: Front Pharmacol. 2013 Nov 28;4:148. doi: 10.3389/fphar.2013.00148 (PMC3842897; doi:10.3389/fphar.2013.00148)
Supplement: Supplementary file 1 [file DataSheet2.PDF]

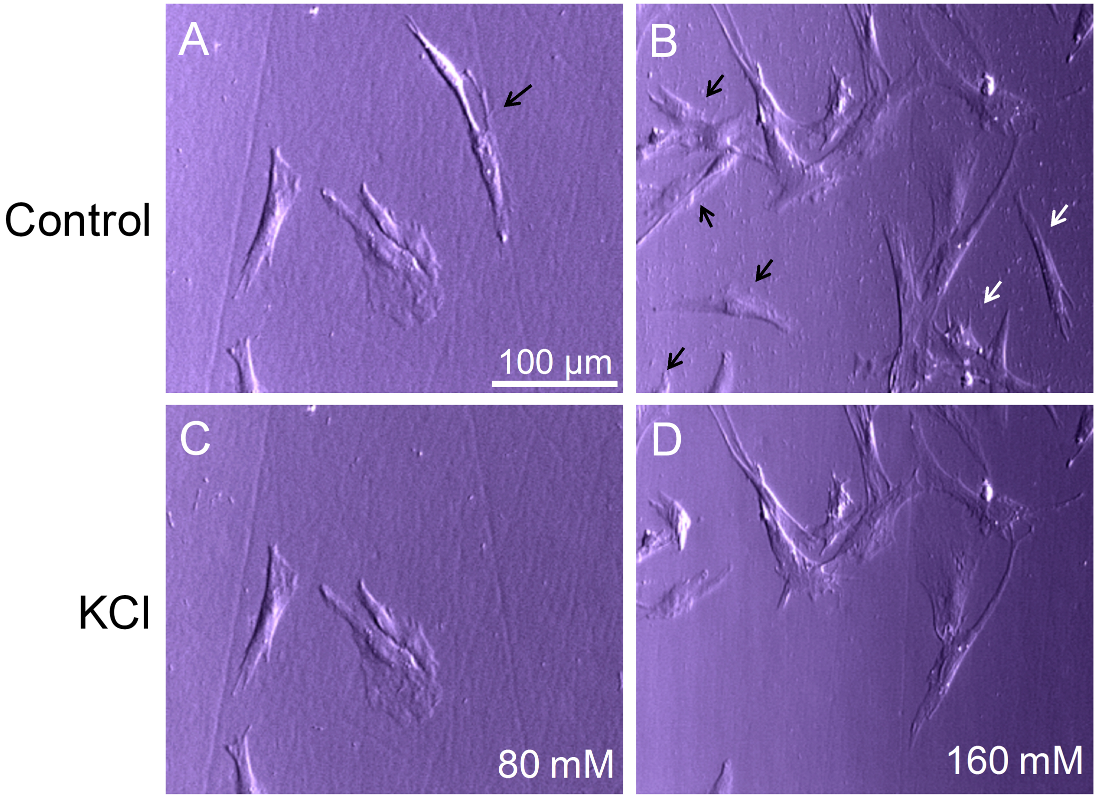

Figure s1: Contractile responses of cultured smooth muscle cells to KCl. Pictures are shown before (control, A, B) and 1 min after exposure to KCl 80 mM (C), and 160 mM (D). Bar = 100 µm

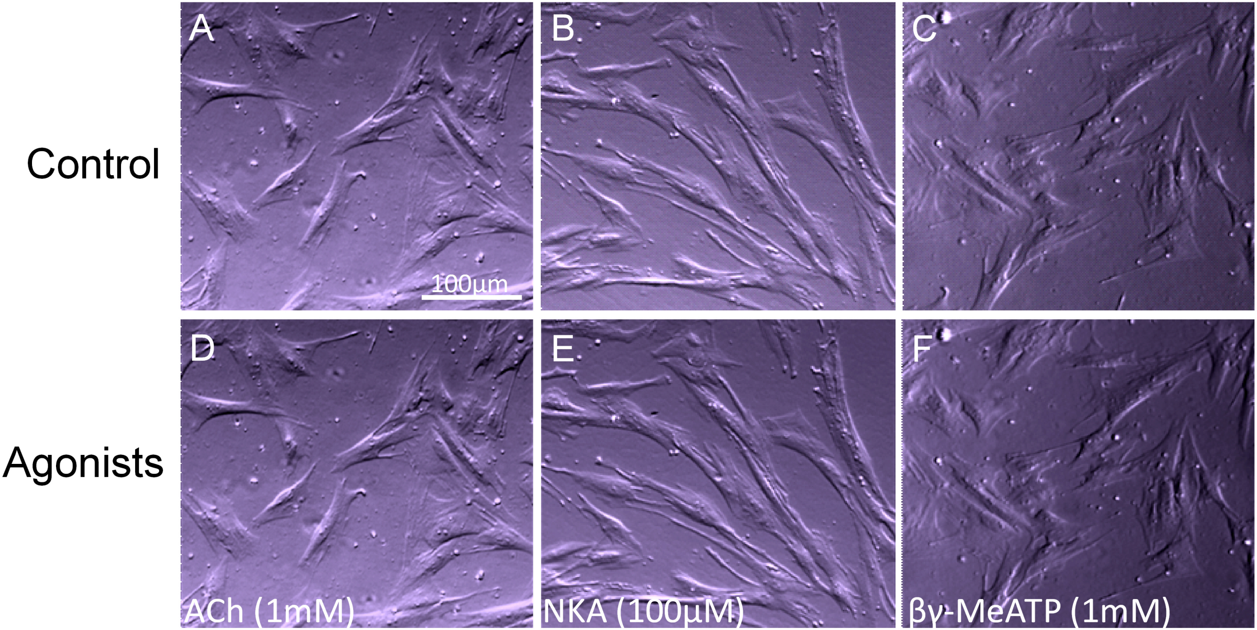

Figure s2: Application of muscarinic, tachykinin and purinergic receptor agonists in cultured muscle cells. Pictures are shown before (control, A-C) and 1 min after ACh (1 mM, D), NKA (100 µM, E) and β,γ-MeATP (1 mM, F) were added to the wells. Bar = 100 µm.

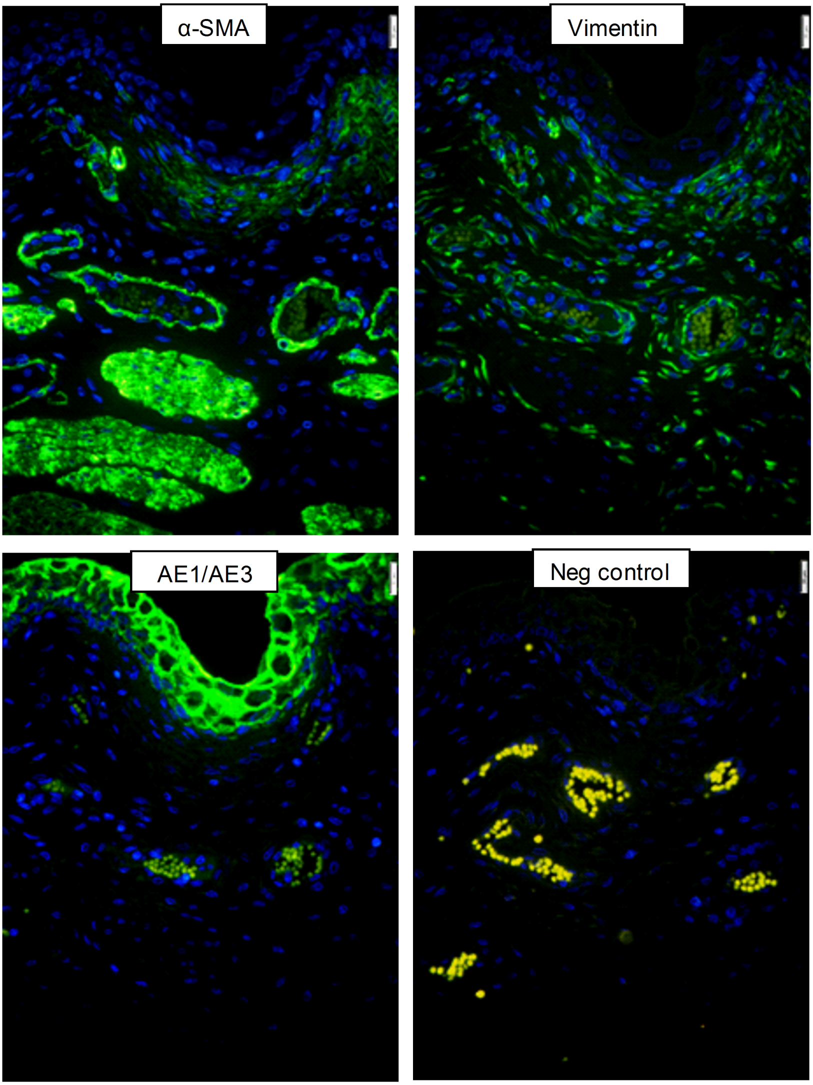

Figure s3: Fluorescent immunostaining of intact segments of porcine bladder for α-SMA, vimentin, AE1/AE3, and negative control (without primary antibody).
